# Supplementary material for: MPG and NPRL3 Polymorphisms Are Associated with Ischemic Stroke Susceptibility and Post-Stroke Mortality
Source: Diagnostics (Basel). 2020 Nov 13;10(11):947. doi: 10.3390/diagnostics10110947 (PMC7696846; doi:10.3390/diagnostics10110947)
Supplement: Supplementary file 1 [file diagnostics-10-00947-s001.zip › diagnostics-954657-SI.11.13/Supple Tables (2020-09-15, RCS).pdf]

Table S1. Information of *NPRL3*, *MPG* polymorphisms for PCR-RFLP and real-time PCR analysis

Gene	rs number	CHR	Position	Primer sequence	Probe sequence	Annealing temperature	Restriction enzyme
NPRL3	rs2541618	ch16	142825	F: 5'- TCC AGG CTG GCT CTT CTA ATC CAC -3'<br>R: 5'- TGA GAT GGG AAC TGG TGG GAG AAC -3'		58ºC	*Sau*96 I
NPRL3	rs75187722	ch16	180529	F: 5'- ATC TGG GTG AAT AGG AGG GTG GGG -3'<br>R: 5'- AAC CCT CCT GTG TGT GGA AGG ACC -3'		60ºC	*Hph*I
MPG	rs2562162	ch16	128179		Oligo 5'- TCA CTG CCC CCC **C**TC TCC CGG CTT C -3'<br>Oligo 5' -TCA CTG CCC CCC **T**TC TCC CGG CTT C -3'	66ºC	
MPG	rs710079	ch16	129223	F: 5′ - ATC TGC TCC CCA GGT CAT GCA G – 3′<br>R: 5′ - GGG TGA CCA TCC TGT GGG TTG T – 3′		60ºC	*Bcc*I

Note: PCR-RFLP, polymorphism chain reaction-restriction fragment length polymorphism; CHR, chromosome.

Table S2. Information of *NPRL3*, *MPG* gene polymorphisms from WES results

Gene	Location	Rs number	*P*-value
MPG	5_prime_UTR	rs76079375	>0.05
MPG	5_prime_UTR	rs2562162	0.00007259
MPG	5_prime_UTR	.	>0.05
MPG	5_prime_UTR	.	>0.05
MPG	5_prime_UTR	rs710079	>0.05
MPG	5_prime_UTR	rs3176380;rs2234890	>0.05
MPG	5_prime_UTR	rs710080	>0.05
MPG	synonymous	rs710081	>0.05
MPG	missense	rs201536549	>0.05
MPG	intron	.	>0.05
NPRL3	intron	rs743725	0.0002432
NPRL3	frameshift	rs57321480;rs397815833	>0.05
NPRL3	intron	rs559166204	>0.05
NPRL3	intron	rs2541618	0.0000468
NPRL3	intron	rs2541616	>0.05
NPRL3	intron	rs808892	0.0003943
NPRL3	intron	rs369659219	>0.05
NPRL3	intron	rs116993855	>0.05
NPRL3	intron	rs148796095	>0.05
NPRL3	intron	.	>0.05
NPRL3	intron	rs138059300	>0.05
NPRL3	splice_region & intron	rs61016911	>0.05
NPRL3	synonymous	rs75187722	0.01976
NPRL3	splice_acceptor & 5_prime_UTR & intron	.	>0.05

Note: NPRL3, nitrogen permease receptor like-3; MPG, N-methylpurine DNA glycosylase.

Table S3. Comparison of baseline characteristics between ischemic stroke patients, ischemic stroke subgroups, and controls

Characteristic	Controls (n=417)	Stroke patients (n=519)	P^a	LAD patients (n=207)	P^a	SVD patients (n=149)	P^a	CE patients (n=53)	P^a
BMI (kg/m2, mean ±SD)	24.37±3.22	24.18±3.11	0.439	24.39±3.07	0.931	23.98±3.16	0.253	23.84±3.27	0.290
HDL-C (mg/dl, mean ±SD)	46.39±13.67	44.58±15.62	**0.021**[b]	43.52±13.26	**0.039**	44.03±13.77	0.127	46.33±13.76	0.975
Homocysteine (μmol/L, mean ±SD)	10.07±4.18	11.21±7.34	**0.005**[b]	11.40±7.95	**0.047**[b]	10.89±5.67	0.085[b]	9.44±3.75	0.294
Folate (nmol/L, mean±SD)	8.83±7.92	7.10±6.15	**< 0.0001**[b]	6.40±4.21	**< 0.0001**[b]	7.07±5.53	**< 0.0001**[b]	9.73±11.53	0.309[b]
Vitamin B12 (pg/ml, mean ±SD)	741.34±662.85	752.10±641.72	0.803	794.03±872.81	0.935[b]	657.32±314.81	0.148[b]	813.58±433.98	**0.013**[b]
Total cholesterol (mg/dl, mean ±SD)	193.12±36.99	190.33±40.40	0.280	193.21±46.28	0.682[b]	189.32±36.23	0.283	180.25±34.59	**0.017**
Triglyceride (mg/dl, mean ±SD)	146.69±89.81	152.82±114.81	0.761[b]	152.10±100.27	0.500	169.29±125.53	0.099[b]	134.02±181.59	**0.007**[b]
PLT ($10^3/\mu\ell$, mean ±SD)	242.70±67.84	244.71±76.82	0.509[b]	251.65±84.05	0.802[b]	239.11±70.40	0.584	233.34±74.61	0.351
PT (sec, mean ±SD)	11.78±0.80	11.82±1.04	0.793[b]	11.78±0.76	1.000	11.68±0.79	0.199	12.07±1.05	0.099[b]
aPTT (sec, mean ±SD)	33.39±18.51	30.57±4.78	**0.043**[b]	30.49±4.82	0.052[b]	30.82±4.70	0.273[b]	30.80±4.27	0.760[b]
Fibrinogen (mg/dl, mean ±SD)	402.27±126.70	425.30±127.15	0.054	431.97±128.17	0.224	398.40±115.24	0.780[b]	453.96±133.29	0.086
Antithrombin III (%, mean ±SD)	94.16±43.02	93.95±17.39	0.078[b]	94.82±15.54	0.056[b]	95.45±20.05	**0.045**[b]	86.28±17.23	0.130[b]
BUN (mg/dl, mean ±SD)	15.81±5.00	16.14±6.39	0.920[b]	15.41±4.85	0.348	15.08±5.14	0.135	18.68±11.04	0.068[b]
Uric Acid (mg/dl, mean ±SD)	4.64±1.47	4.70±1.52	0.561	4.67±1.42	0.811	4.62±1.36	0.905	4.54±1.56	0.644

Note: SD, standard deviation; BMI, body mass index; HDL-C, high density lipoprotein cholesterol; PLT, platelet; PT, prothrombin time; aPTT, activated partial thromboplastin time; BUN, blood urea nitrogen; LAD, large artery disease; SVD, small vessel disease; CE, cardioembolism.

[a] *P*-values were calculated by two-sided t-test for continuous variables and chi-square test for categorical variables.

[b] *P*-values were calculated by Mann-Whitney-test for continuous variables.

***P*-values< 0,05 are bold**

Table S4. Stratified analysis of *NPRL3* genotypes and characteristics of ischemic stroke among individual risk factors

Characteristics	*NPRL3* rs2541618 TT		*NPRL3* rs2541618 CT+TT		*NPRL3* rs75187722 GA		*NPRL3* rs75187722 GA+AA	
	AOR(95% CI)*	*P*	AOR(95% CI)*	*P*	AOR(95% CI)*	*P*	AOR(95% CI)*	*P*
Age (936)								
<63	1.757 (0.853 - 3.620)	0.126	1.436 (0.951 - 2.167)	0.085	0.605 (0.350 - 1.044)	0.071	0.589 (0.343 - 1.014)	0.056
≥63	1.412 (0.710 - 2.810)	0.325	1.048 (0.723 - 1.519)	0.805	0.762 (0.468 - 1.241)	0.275	0.794 (0.493 - 1.281)	0.345
Sex (936)								
Male	1.858 (0.913 - 3.782)	0.088	**1.581 (1.033 - 2.421)**	**0.035**	**0.544 (0.310 - 0.957)**	**0.035**	**0.544 (0.310 - 0.957)**	**0.035**
Female	1.339 (0.673 - 2.667)	0.406	1.012 (0.708 - 1.448)	0.947	0.815 (0.504 - 1.318)	0.404	0.825 (0.517 - 1.316)	0.419
Hypertension (936)								
No	1.169 (0.586 - 2.333)	0.657	1.060 (0.714 - 1.574)	0.773	0.993 (0.598 - 1.650)	0.978	0.993 (0.598 - 1.650)	0.978
Yes	**2.109 (1.005 - 4.427)**	**0.049**	1.375 (0.944 - 2.002)	0.097	**0.529 (0.321 - 0.872)**	**0.012**	**0.550 (0.339 - 0.893)**	**0.016**
Diabetes mellitus (936)								
No	1.403 (0.824 - 2.392)	0.213	1.154 (0.853 - 1.561)	0.353	0.685 (0.454 - 1.033)	0.071	0.681 (0.454 - 1.020)	0.062
Yes	2.782 (0.702 - 11.017)	0.145	1.651 (0.863 - 3.160)	0.130	0.766 (0.349 - 1.683)	0.507	0.838 (0.385 - 1.822)	0.656
Hyperlipidemia (936)								
No	1.378 (0.805 - 2.361)	0.242	1.180 (0.862 - 1.616)	0.301	**0.599 (0.394 - 0.909)**	**0.016**	**0.594 (0.394 - 0.897)**	**0.013**
Yes	3.132 (0.786 - 12.479)	0.106	1.367 (0.791 - 2.362)	0.262	1.233 (0.574 - 2.651)	0.591	1.318 (0.619 - 2.805)	0.474
Smoking (930)								
No	1.771 (0.918 - 3.415)	0.088	1.225 (0.873 - 1.719)	0.241	0.775 (0.495 - 1.216)	0.268	0.767 (0.493 - 1.193)	0.240
Yes	1.504 (0.707 - 3.200)	0.289	1.181 (0.745 - 1.871)	0.479	**0.535 (0.287 - 0.998)**	**0.049**	0.568 (0.307 - 1.052)	0.072
HDL-c (671)								
≥40(M)/50(F)	1.083 (0.445 - 2.634)	0.860	0.945 (0.570 - 1.568)	0.827	0.510 (0.238 - 1.094)	0.084	0.510 (0.238 - 1.094)	0.084
<40(M)/50(F)	1.354 (0.556 - 3.295)	0.504	1.453 (0.877 - 2.405)	0.147	0.589 (0.324 - 1.070)	0.082	0.623 (0.345 - 1.127)	0.118
Folate * (927)								
>3.54 nmol/L	1.387 (0.808 - 2.381)	0.235	1.176 (0.874 - 1.582)	0.285	0.917 (0.618 - 1.359)	0.665	0.910 (0.617 - 1.341)	0.633
≤3.54 nmol/L	2.949 (0.580 - 15.003)	0.193	1.408 (0.613 - 3.237)	0.420	**0.137 (0.048 - 0.386)**	**0.0002**	**0.142 (0.051 - 0.399)**	**0.0002**
Homocysteine † (931)								
<13.7 μmol/L	1.546 (0.917 - 2.605)	0.102	1.257 (0.936 - 1.687)	0.128	0.736 (0.493 - 1.098)	0.133	0.737 (0.497 - 1.093)	0.129
≥13.7 μmol/L	1.454 (0.295 - 7.171)	0.646	0.930 (0.432 - 2.001)	0.852	0.537 (0.217 - 1.329)	0.179	0.578 (0.237 - 1.411)	0.229
Platelet † (927)								
<305 $10^3/\mu\ell$	1.375 (0.812 - 2.330)	0.236	1.138 (0.847 - 1.531)	0.391	0.918 (0.610 - 1.381)	0.680	0.906 (0.606 - 1.355)	0.630
≥305 $10^3/\mu\ell$	3.847 (0.859 - 17.218)	0.078	1.814 (0.880 - 3.742)	0.107	**0.291 (0.121 - 0.701)**	**0.006**	**0.322 (0.137 - 0.757)**	**0.009**
PT * (801)								
>11.00 sec	1.197 (0.690 - 2.075)	0.523	1.157 (0.834 - 1.605)	0.382	**0.577 (0.381 - 0.874)**	**0.009**	**0.560 (0.371 - 0.844)**	**0.006**
≤11.00 sec	3.859 (0.627 - 23.766)	0.145	1.853 (0.851 - 4.037)	0.120	1.093 (0.365 - 3.280)	0.874	1.241 (0.426 - 3.614)	0.692
aPTT * (801)								
>26.30 sec	1.678 (0.928 - 3.035)	0.087	1.233 (0.890 - 1.709)	0.207	**0.585 (0.385 - 0.889)**	**0.012**	**0.588 (0.389 - 0.890)**	**0.012**
≤26.30 sec	0.568 (0.164 - 1.973)	0.373	1.559 (0.698 - 3.481)	0.279	0.744 (0.238 - 2.320)	0.610	0.666 (0.223 - 1.988)	0.466
Fibrinogen † (626)								
<537 mg/dl	1.685 (0.784 - 3.621)	0.181	1.334 (0.886 - 2.007)	0.167	0.692 (0.411 - 1.165)	0.166	0.720 (0.429 - 1.208)	0.213
≥537 mg/dl	0.948 (0.196 - 4.590)	0.947	1.280 (0.413 - 3.966)	0.669	**0.176 (0.040 - 0.767)**	**0.021**	**0.176 (0.040 - 0.767)**	**0.021**

Note: AOR, adjusted odds ratio; 95% CI, 95% confidence interval; HDL-c, high density lipoprotein cholesterol; PT, prothrombin time; aPTT, activated partial thromboplastin time; NPRL3, nitrogen permease receptor like-3. * The adjusted odds ratio on the basis of risk factors, such as age, gender, hypertension, diabetes mellitus, hyperlipidemia, smoking. *P*-values <0.05 are bold. The number of each subgroup is located next to each subgroup.

† Folate 3.54nmol/L, PT 11.00 sec, and aPTT 26.30 sec were lower 15% cut-off each level in ischemic stroke patients and controls.

‡ Homocysteine 13.7 μmol/L, Platelet 305 103/μℓ, and fibrinogen 537 mg/dl were upper 15% cut-off each level in ischemic stroke patients and controls.

Table S5. Stratified analysis of *MPG* genotypes and characteristics of ischemic stroke among individual risk factors

Characteristics	*MPG* rs2562162 CT		*MPG* rs2562162 CT+TT		*MPG* rs710079 CT		*MPG* rs710079 CT+TT	
	AOR(95% CI)*	*P*	AOR(95% CI)*	*P*	AOR(95% CI)*	*P*	AOR(95% CI)*	*P*
Age (936)								
<63	1.339 (0.876 - 2.045)	0.177	1.435 (0.952 - 2.164)	0.085	0.938 (0.592 - 1.486)	0.784	0.903 (0.573 - 1.424)	0.661
≥63	1.255 (0.835 - 1.885)	0.275	1.157 (0.792 - 1.689)	0.451	0.950 (0.628 - 1.436)	0.808	0.915 (0.612 - 1.367)	0.664
Sex (936)								
Male	1.407 (0.899 - 2.203)	0.135	1.461 (0.953 - 2.239)	0.082	0.931 (0.575 - 1.508)	0.771	0.866 (0.540 - 1.388)	0.549
Female	1.229 (0.839 - 1.800)	0.289	1.168 (0.814 - 1.676)	0.399	0.924 (0.618 - 1.380)	0.698	0.914 (0.618 - 1.352)	0.652
Hypertension (936)								
No	1.357 (0.893 - 2.063)	0.153	1.415 (0.948 - 2.110)	0.089	1.201 (0.780 - 1.847)	0.406	1.093 (0.715 - 1.672)	0.680
Yes	1.191 (0.802 - 1.769)	0.387	1.127 (0.774 - 1.640)	0.534	0.779 (0.508 - 1.195)	0.252	0.798 (0.526 - 1.210)	0.288
Diabetes mellitus (936)								
No	1.188 (0.862 - 1.636)	0.293	1.184 (0.873 - 1.605)	0.277	0.859 (0.614 - 1.202)	0.376	0.829 (0.596 - 1.154)	0.266
Yes	1.875 (0.945 - 3.719)	0.072	1.830 (0.954 - 3.512)	0.069	1.728 (0.757 - 3.946)	0.194	1.570 (0.728 - 3.388)	0.250
Hyperlipidemia (936)								
No	1.373 (0.980 - 1.923)	0.065	1.340 (0.976 - 1.839)	0.071	0.863 (0.608 - 1.226)	0.412	0.821 (0.582 - 1.159)	0.262
Yes	1.108 (0.634 - 1.936)	0.719	1.113 (0.646 - 1.917)	0.700	1.321 (0.701 - 2.489)	0.390	1.309 (0.709 - 2.418)	0.389
Smoking (930)								
No	1.283 (0.896 - 1.838)	0.174	1.238 (0.880 - 1.741)	0.221	1.004 (0.683 - 1.477)	0.983	0.952 (0.654 - 1.387)	0.799
Yes	1.302 (0.800 - 2.119)	0.289	1.341 (0.845 - 2.126)	0.213	0.795 (0.477 - 1.327)	0.380	0.779 (0.469 - 1.292)	0.333
HDL-c (671)								
≥40(M)/50(F)	1.345 (0.790 - 2.289)	0.276	1.358 (0.819 - 2.253)	0.236	0.997 (0.562 - 1.769)	0.992	0.966 (0.547 - 1.706)	0.904
<40(M)/50(F)	1.311 (0.763 - 2.252)	0.328	1.276 (0.765 - 2.127)	0.350	**0.571 (0.334 - 0.976)**	**0.040**	**0.589 (0.347 - 0.999)**	**0.050**
Folate * (927)								
>3.54 nmol/L	1.277 (0.936 - 1.741)	0.123	1.235 (0.917 - 1.663)	0.165	1.026 (0.733 - 1.437)	0.879	0.969 (0.698 - 1.347)	0.852
≤3.54 nmol/L	1.391 (0.538 - 3.598)	0.496	1.555 (0.654 - 3.698)	0.318	0.635 (0.257 - 1.572)	0.326	0.714 (0.293 - 1.738)	0.458
Homocysteine † (931)								
<13.7 μmol/L	**1.438 (1.054 - 1.960)**	**0.022**	**1.455 (1.083 - 1.956)**	**0.013**	0.909 (0.652 - 1.268)	0.573	0.864 (0.624 - 1.195)	0.377
≥13.7 μmol/L	0.536 (0.236 - 1.215)	0.135	0.496 (0.229 - 1.070)	0.075	1.347 (0.590 - 3.076)	0.480	1.407 (0.618 - 3.203)	0.415
Platelet † (927)								
<305 10^3/μℓ	1.159 (0.846 - 1.586)	0.358	1.140 (0.846 - 1.535)	0.390	1.032 (0.738 - 1.443)	0.854	0.996 (0.717 - 1.383)	0.980
≥305 10^3/μℓ	**2.137 (1.007 - 4.533)**	**0.048**	**2.278 (1.106 - 4.691)**	**0.026**	0.691 (0.317 - 1.505)	0.352	0.654 (0.303 - 1.412)	0.279
PT * (801)								
>11.00 sec	1.154 (0.815 - 1.635)	0.420	1.121 (0.808 - 1.555)	0.496	0.857 (0.598 - 1.229)	0.401	0.813 (0.571 - 1.158)	0.251
≤11.00 sec	1.976 (0.864 - 4.518)	0.106	1.798 (0.812 - 3.980)	0.148	1.349 (0.550 - 3.309)	0.513	1.349 (0.559 - 3.252)	0.506
aPTT * (801)								
>26.30 sec	1.125 (0.798 - 1.587)	0.502	1.121 (0.809 - 1.555)	0.493	0.908 (0.630 - 1.307)	0.602	0.857 (0.599 - 1.224)	0.396
≤26.30 sec	2.228 (0.916 - 5.417)	0.077	1.662 (0.731 - 3.775)	0.225	0.998 (0.424 - 2.348)	0.996	0.969 (0.419 - 2.239)	0.941
Fibrinogen † (626)								
< 537 mg/dl	1.552 (0.999 - 2.413)	0.051	**1.598 (1.050 - 2.433)**	**0.029**	0.839 (0.535 - 1.314)	0.443	0.834 (0.535 - 1.298)	0.421
≥ 537 mg/dl	1.314 (0.408 - 4.232)	0.647	1.259 (0.419 - 3.778)	0.682	0.601 (0.190 - 1.901)	0.386	0.601 (0.190 - 1.901)	0.386

Note: AOR, adjusted odds ratio; 95% CI, 95% confidence interval; HDL-c, high density lipoprotein cholesterol; PT, prothrombin time; aPTT, activated partial thromboplastin time; MPG, N-methylpurine DNA glycosylase. * The adjusted odds ratio on the basis of risk factors, such as age, gender, hypertension, diabetes mellitus, hyperlipidemia, smoking. *P*-values <0.05 are bold. The number of each subgroup is located next to each subgroup.

† Folate 3.54nmol/L, PT 11.00 sec, and aPTT 26.30 sec were lower 15% cut-off each level in ischemic stroke patients and controls.

‡ Homocysteine 13.7 μmol/L, Platelet 305 103/μℓ, and fibrinogen 537 mg/dl were upper 15% cut-off each level in ischemic stroke patients and controls.

Table S6. Ischemic stroke incidence by interaction analysis between *NPRL3*, *MPG* genotypes and environmental factors

Characteristics	*NPRL3* rs2541618 CC	*NPRL3* rs2541618 CT+TT	*NPRL3* rs75187722 GG	*NPRL3* rs75187722 GA+AA	*MPG* rs2562162 CC	*MPG* rs2562162 CT+TT	*MPG* rs710079 CC	*MPG* rs710079 CT+TT
HDL-c (671)								
≥40(M)/50(F)	1.000 (reference)	1.089 (0.758 - 1.565)	1.000 (reference)	**0.508 (0.297 - 0.870)**	1.000 (reference)	**1.449 (1.007 - 2.085)**	1.000 (reference)	1.078 (0.718 - 1.619)
<40(M)/50(F)	**4.529 (2.900 - 7.074)**	**6.364 (4.103 - 9.869)**	**5.541 (3.880 - 7.912)**	**3.699 (2.099 - 6.520)**	**5.718 (3.754 - 8.709)**	**7.330 (4.564 - 11.773)**	**6.449 (4.384 - 9.487)**	**3.929 (2.412 - 6.400)**
Folate * (927)								
>3.54 nmol/L	1.000 (reference)	1.187 (0.883 - 1.596)	1.000 (reference)	0.911 (0.619 - 1.340)	1.000 (reference)	1.254 (0.932 - 1.686)	1.000 (reference)	0.953 (0.687 - 1.322)
≤3.54 nmol/L	**3.235 (1.739 - 6.019)**	**4.866 (2.527 - 9.370)**	**5.427 (3.151 - 9.347)**	0.696 (0.282 - 1.717)	**3.347 (1.892 - 5.919)**	**5.601 (2.651 - 11.836)**	**3.925 (2.270 - 6.785)**	**2.622 (1.247 - 5.517)**
Homocysteine † (931)								
<13.7 μmol/L	1.000 (reference)	1.261 (0.940 - 1.691)	1.000 (reference)	0.738 (0.499 - 1.092)	1.000 (reference)	**1.463 (1.088 - 1.965)**	1.000 (reference)	0.860 (0.621 - 1.190)
≥13.7 μmol/L	1.727 (0.976 - 3.056)	**1.839 (1.057 - 3.201)**	**1.639 (1.041 - 2.581)**	0.857 (0.391 - 1.875)	**2.365 (1.374 - 4.071)**	1.301 (0.731 - 2.318)	1.281 (0.797 - 2.059)	1.747 (0.882 - 3.462)
Platelet † (927)								
<305 10^3/μℓ	1.000 (reference)	1.140 (0.849 - 1.530)	1.000 (reference)	0.870 (0.585 - 1.296)	1.000 (reference)	1.158 (0.861 - 1.557)	1.000 (reference)	0.991 (0.716 - 1.373)
≥305 10^3/μℓ	0.670 (0.379 - 1.187)	1.205 (0.711 - 2.042)	1.170 (0.749 - 1.829)	**0.356 (0.163 - 0.776)**	0.658 (0.385 - 1.123)	1.348 (0.770 - 2.357)	1.033 (0.650 - 1.643)	0.662 (0.342 - 1.281)
PT * (801)								
>11.00 sec	1.000 (reference)	1.113 (0.831 - 1.491)	1.000 (reference)	**0.666 (0.454 - 0.976)**	1.000 (reference)	1.182 (0.882 - 1.585)	1.000 (reference)	0.900 (0.653 - 1.240)
≤11.00 sec	1.067 (0.606 - 1.881)	**2.192 (1.241 - 3.872)**	1.319 (0.855 - 2.035)	1.579 (0.608 - 4.099)	1.201 (0.690 - 2.088)	**2.071 (1.164 - 3.682)**	1.364 (0.858 - 2.170)	1.564 (0.744 - 3.290)
aPTT * (801)								
>26.30 sec	1.000 (reference)	1.176 (0.879 - 1.573)	1.000 (reference)	**0.679 (0.463 - 0.996)**	1.000 (reference)	1.215 (0.907 - 1.627)	1.000 (reference)	0.910 (0.660 - 1.257)
≤26.30 sec	1.190 (0.668 - 2.121)	1.731 (0.971 - 3.086)	1.273 (0.812 - 1.996)	1.255 (0.489 - 3.222)	1.210 (0.696 - 2.105)	1.832 (0.996 - 3.372)	1.312 (0.801 - 2.149)	1.292 (0.648 - 2.579)
Fibrinogen † (626)								
< 537 mg/dl	1.000 (reference)	1.204 (0.904 - 1.603)	1.000 (reference)	0.804 (0.553 - 1.168)	1.000 (reference)	1.272 (0.954 - 1.696)	1.000 (reference)	0.982 (0.717 - 1.346)
≥ 537 mg/dl	**2.690 (1.276 - 5.670)**	**3.464 (1.698 - 7.065)**	**3.535 (1.972 - 6.335)**	0.702 (0.223 - 2.209)	**2.926 (1.483 - 5.770)**	**3.460 (1.580 - 7.578)**	**3.481 (1.838 - 6.595)**	1.716 (0.727 - 4.049)

Note: NPRL3, nitrogen permease receptor like-3; MPG, N-methylpurine DNA glycosylase; PT, prothrombin time; aPTT, activated partial thromboplastin time. *P*-values <0.05 are bold. The number of each subgroup is located next to each subgroup.

* Folate 3.54nmol/L, PT 11.00 sec, and aPTT 26.30 sec were lower 15% cut-off each level in ischmic stroke patients and controls.

† Homocystein 13.7 umol/L, Platelet 305 10^3/μℓ, and Fibrinogen 537 mg/dl were upper 15% cut-off each level in ischmic stroke patients and controls.

Table S7. Clinical variables of ischemic stroke patients and subtypes stratified according to *NPRL3* and *MPG* polymorphisms

Genotypes	PLT (10^3/μL)		Uric acid (mg/dL)		LAD patients Platelets (10^3/μL)		LAD patients Uric acid (mg/dL)		LAD patients HDL-c (mg/dL)		SVD patients Fibrinogen (mg/dL)	
	Mean±SD	P^*	Mean±SD	P^*	Mean±SD	P^*	Mean±SD	P^*	Mean±SD	P^*	Mean±SD	P^*
NPRL3 rs2541618C>T												
CC	238.37±81.92	0.256	4.58±1.50	0.455	245.99±100.50	0.682	4.46±1.39	0.157	45.68±16.13	0.110	393.32±112.43	0.103
CT	249.98±71.92		4.76±1.57		255.96±66.07		4.83±1.50		41.52±10.56		387.55±113.65	
TT	247.66±75.39		4.90±1.35		257.40±75.36		4.87±1.18		42.55±8.42		450.26±120.70	
Dominant (CC vs CT+TT)	249.54±72.47	0.101	4.79±1.53	0.114	256.27±67.87	**0.030**†	4.84±1.43	0.055	41.76±10.08	**0.038**	401.57±117.51	0.684
Recessive (CC+CT vs TT)	247.66±75.39	0.761	4.90±1.35	0.282	257.40±75.36	0.646†	4.87±1.18	0.444	42.55±8.42	0.696	450.26±120.70	**0.034**
NPRL3 rs75187722G>A												
GG	245.19±78.12	0.916	4.70±1.49	**0.028**	253.51±83.97	0.423	4.76±1.46	**0.023**†	43.31±10.28	0.364†	397.16±118.19	0.922
GA	241.61±70.88		4.58±1.58		239.79±85.13		4.08±0.94		44.98±26.14		407.14±93.19	
AA	251.75±50.07		6.65±2.67		-		-		-		429.00±0.00	
Dominant (GG vs GA+AA)	242.11±69.79	0.741	4.68±1.68	0.927	239.79±85.13	0.152†	4.08±0.94	**0.023**†	44.98±26.14	0.364†	408.60±89.98	0.718
Recessive (GG+GA vs AA)	251.75±50.07	0.854	6.65±2.67	0.098†	-		-		-		429.00±0.00	0.791
MPG rs2562162C>T												
CC	237.17±76.06	0.071	4.74±1.50	0.710	242.25±91.32	0.247	4.60±1.28	0.586	43.49±15.36	0.858	391.96±110.84	0.702
CT	253.07±75.91		4.63±1.53		260.11±72.14		4.78±1.61		43.24±11.10		406.78±120.93	
TT	249.89±84.18		4.76±1.54		269.00±94.22		4.48±1.24		45.37±8.72		383.83±110.54	
Dominant (CC vs CT+TT)	252.61±76.97	**0.022**	4.65±1.53	0.499	261.33±75.07	0.103	4.74±1.56	0.875†	43.55±10.78	0.977	403.50±119.08	0.561
Recessive (CC+CT vs TT)	249.89±84.18	0.675	4.76±1.54	0.789	269.00±94.22	0.425	4.48±1.24	0.607	45.37±8.72	0.589	383.83±110.54	0.664
MPG rs710079C>T												
CC	245.56±78.74	0.629	4.69±1.48	0.937	254.09±86.26	0.316	4.74±1.50	0.342	43.06±10.48	0.406	395.93±118.45	0.874
CT	243.76±72.65		4.71±1.62		247.13±77.30		4.49±1.17		45.11±19.31		406.91±105.49	
TT	217.71±46.82		4.87±1.54		132.00±0.00		3.30±0.00		30.80±0.00		429.00±0.00	
Dominant (CC vs CT+TT)	242.47±71.71	0.684	4.72±1.62	0.809	245.07±78.12	0.595†	4.47±1.17	0.227	44.83±19.22	0.413	407.67±103.67	0.628
Recessive (CC+CT vs TT)	217.71±46.82	0.350	4.87±1.54	0.758	132.00±0.00	0.090†	3.30±0.00	0.336	30.80±0.00	0.337	429.00±0.00	0.791

Note: ANOVA, analysis of variance; HDL-c, high density lipoprotein cholesterol; LAD, large artery disease; SVD, small vessel disease; CE, cardioembolism; SD, standard deviation; NPRL3, nitrogen permease receptor like-3; MPG, N-methylpurine DNA glycosylase.

*Calculated using ANOVA. †Calculated using the Kruskal-Wallis test. *P*-values< 0,05 are bold

Table S8. Results of stepwise Cox regression analysis for ischemic stroke survival

Covariate	β	SEM	HR (95% CI)	*P**
MPG rs2562162 CC vs CT in SVD group				
Age	0.078	0.026	1.081 (1.028 - 1.137)	**0.003**
MPG rs2562162 CC vs CT+TT in SVD group				
Age	0.078	0.024	1.082 (1.032 - 1.133)	**0.001**
MPG rs2562162 CC vs CT+TT in SVD group/with hypertension				
Genetic variant (CC vs CT+TT)	1.686	0.810	0.185 (0.038 - 0.899)	**0.037**
Age	0.100	0.035	1.105 (1.032 - 1.183)	**0.001**
MPG rs710079 CC vs CT with diabetes mellitus				
Genetic variant (CC vs CT)	0.788	0.381	2.200 (1.046 - 4.625)	**0.039**
Age	0.052	0.019	1.054 (1.016 - 1.093)	**0.005**
NPRL3 rs2541618 CC vs CT+TT with hyperlipidemia				
Genetic variant (CC vs CT+TT)	1.134	0.556	3.107 (1.051 - 9.183)	**0.041**
Age	0.076	0.019	1.079 (1.040 - 1.120)	**<0.001**
MPG rs710079 CC vs CT in male				
Genetic variant (CC vs CT)	0.825	0.370	2.282 (1.109 - 4.696)	**0.026**
Age	0.075	0.015	1.078 (1.047 - 1.109)	**<0.0001**

Note: SEM, standard error of the mean; HR, hazard ratio; CI, confidence interval; NPRL3, nitrogen permease receptor like-3; MPG, N-methylpurine DNA glycosylase; SVD, small vessel disease.

**P*-value calculated by Cox proportional-hazards regression based on stepwise method. *P*-values< 0,05 are bold

Table S9. Statistical power to detect various genetic associations in the present case-control study

Characteristics	Table	AOR (95% CI)	Statistical power (%)
NPRL3 rs75187722 Dominant model	Table 2 (LAD)	0.599 (0.370 - 0.968)	51.44
NPRL3 rs2541618TT	Table 2 (SVD)	2.406 (1.225 - 4.725)	54.13
NPRL3 rs2541618 Dominant model	Table 2 (SVD)	1.524 (1.019 - 2.279)	61.58
NPRL3 rs75187722GA	Table 2 (SVD)	0.474 (0.261 - 0.860)	53.77
NPRL3 rs75187722 Dominant model	Table 2 (SVD)	0.495 (0.277 - 0.886)	50.62
MPG rs2562162CT	Table 2 (SVD)	1.569 (1.038 - 2.372)	78.95
MPG rs2562162 Dominant model	Table 2 (SVD)	1.589 (1.069 - 2.363)	80.70

Note: AOR, adjust odds ratio; 95% CI, 95% confidence interval.

Table S10. The results of meta-analysis restricted to Europeans in the MEGASTROKE GWAS loci of *NPRL3* and *MPG* genes polymorphisms

			Ischemic stroke patients & controls					LAD patients & controls				SVD patients & controls				CE patients & controls			
SNP	Chr	Gene	Risk allele	MAF	Effect	StdErr	*P*-value	MAF	Effect	StdErr	*P*-value	MAF	Effect	StdErr	*P*-value	MAF	Effect	StdErr	*P*-value
rs2541618	16	NPRL3	T	0.142	0.019	0.015	0.203	0.146	0.007	0.038	0.855	0.141	0.078	0.035	**0.024**	0.144	0.025	0.029	0.400
rs75187722	16	NPRL3	A	0.016	-0.069	0.057	0.222	0.015	-0.097	0.158	0.537	0.016	0.133	0.142	0.350	0.017	-0.123	0.109	0.257
rs2562162	16	MPG	T	0.134	0.033	0.019	0.080	0.137	0.078	0.047	0.093	0.133	0.084	0.042	**0.045**	0.136	0.041	0.037	0.271
rs710079	16	MPG	T	0.017	0.035	0.052	0.509	0.018	0.016	0.141	0.913	0.017	0.101	0.121	0.407	0.018	0.031	0.103	0.764

Note: GWAS, genome-wide association study; SNP, single nucleotide polymorphism; Chr, chromosome; MAF, minor allele frequency; StdErr, standard error; NPRL3, nitrogen permease receptor like-3; MPG, N-methylpurine DNA glycosylase; LAD, large artery disease; SVD, small vessel disease; CE, cardioembolism. *P*-values< 0,05 are bold
