# Supplementary material for: MPG and NPRL3 Polymorphisms Are Associated with Ischemic Stroke Susceptibility and Post-Stroke Mortality
Source: Diagnostics (Basel). 2020 Nov 13;10(11):947. doi: 10.3390/diagnostics10110947 (PMC7696846; doi:10.3390/diagnostics10110947)
Supplement: Supplementary file 1 [file diagnostics-10-00947-s001.zip › Supple Figures.docx]

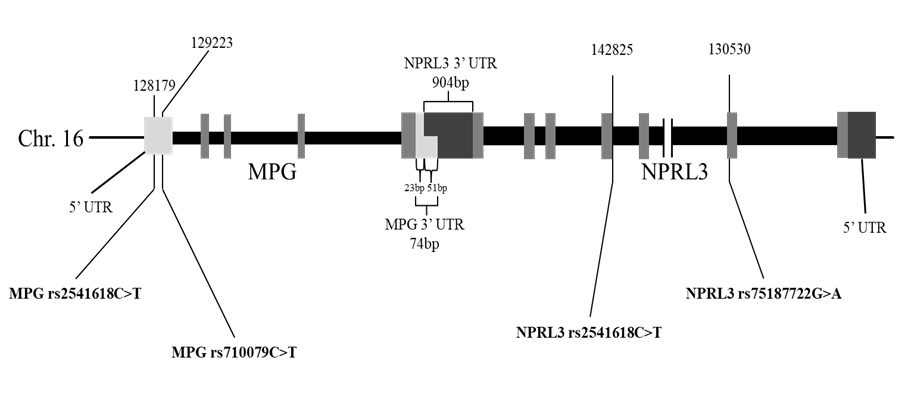


**Figure S1.** Information of *MPG* and *NPRL3* gene polymorphisms position number in chromosome 16. The whole 3’UTR length of *MPG* and *NPRL3* gene is 927bp. Part of *MPG* 3’UTR (74bp) share with *NPRL3* 3’UTR (904bp) and the sharing 3’UTR length is 51bp.


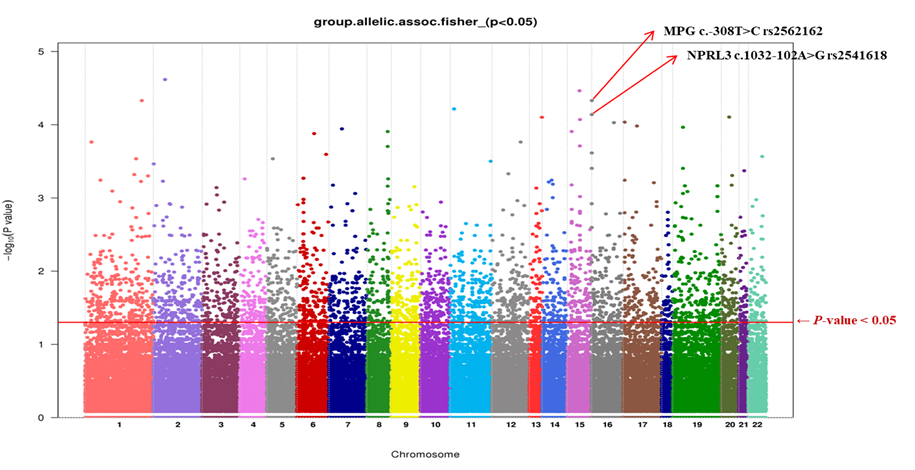


**Figure S2. Information of *MPG* and *NPRL3* gene polymorphism in Manhattan plot of WES analysis.** The significant SNPs were filtered by the significant criteria satisfying *P*<0.05 for Fisher’s exact test, and *MPG* and *NPRL3* gene polymorphisms were selected by Fisher’s exact test (*P* = upper 0.2%).


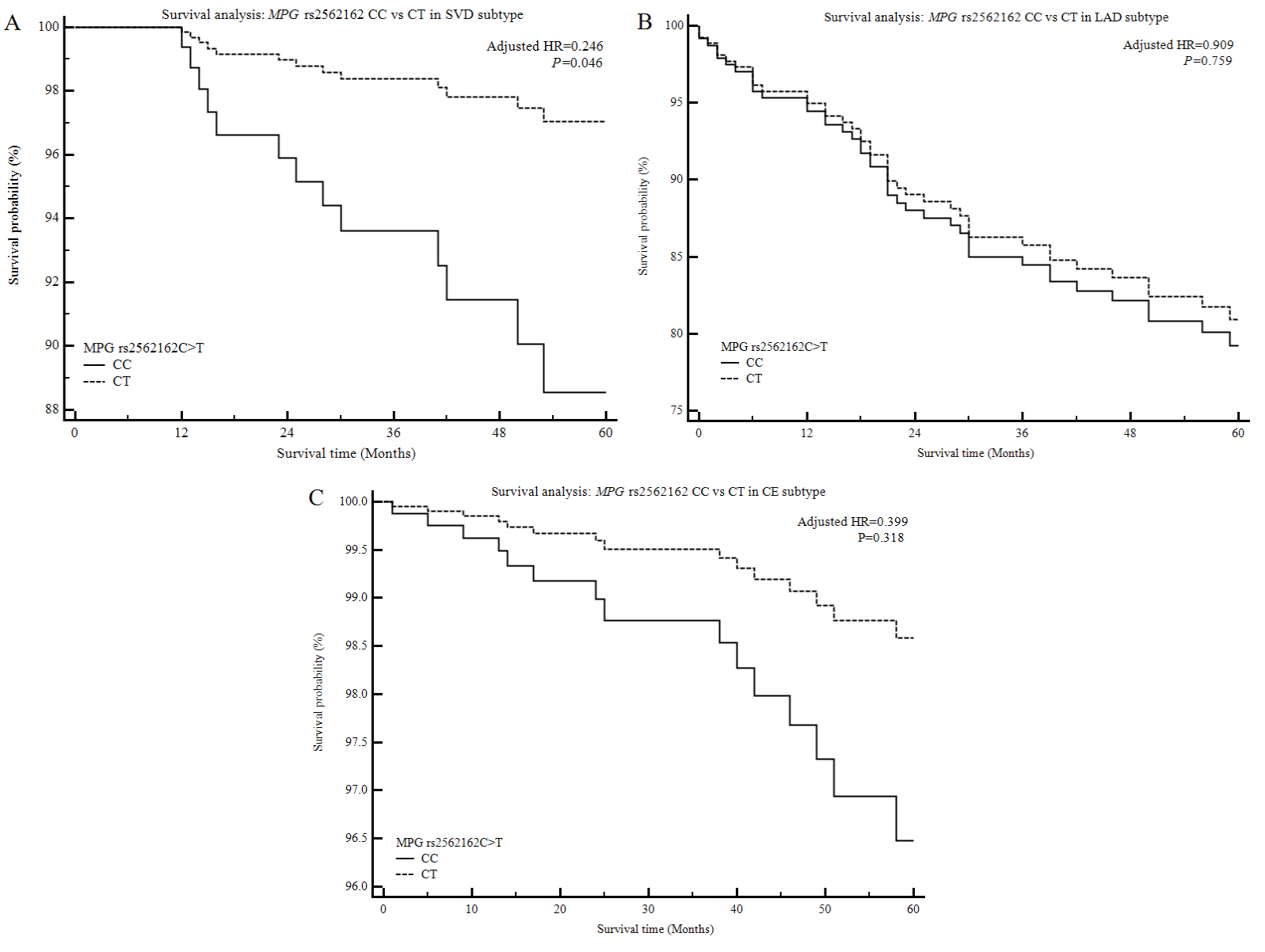


**Figure S3.** Survival plot of a Cox proportional hazards model with *MPG* rs2562162C>T in ischemic stroke. Survival probability of ischemic stroke patients grouped by (A) small vessel disease (SVD), (B) large artery disease (LAD), and (C) cardioembolism (CE) subtype based on *MPG* rs2562162CC vs. *MPG* rs2562162CT genotypes. Note to Figure S2: HR, hazard ratio
