# Supplementary material for: MPG and NPRL3 Polymorphisms Are Associated with Ischemic Stroke Susceptibility and Post-Stroke Mortality
Source: Diagnostics (Basel). 2020 Nov 13;10(11):947. doi: 10.3390/diagnostics10110947 (PMC7696846; doi:10.3390/diagnostics10110947)
Supplement: Supplementary file 1 [file diagnostics-10-00947-s001.zip › Supple Tables (2020-09-15, RCS).docx]

| **Table S1.** Information of *NPRL3*, *MPG* polymorphisms for PCR-RFLP and real-time PCR analysis | | | | | | | |
| --- | --- | --- | --- | --- | --- | --- | --- |
| Gene | rs number | CHR | Position | Primer sequence | Probe sequence | Annealing  temperature | Restriction  enzyme |
| *NPRL3* | rs2541618 | ch16 | 142825 | F: 5'- TCC AGG CTG GCT CTT CTA ATC CAC -3'  R: 5'- TGA GAT GGG AAC TGG TGG GAG AAC -3' |  | 58ºC | *Sau*96 I |
| *NPRL3* | rs75187722 | ch16 | 180529 | F: 5'- ATC TGG GTG AAT AGG AGG GTG GGG -3'  R: 5'- AAC CCT CCT GTG TGT GGA AGG ACC -3' |  | 60ºC | *Hph*I |
| *MPG* | rs2562162 | ch16 | 128179 |  | Oligo 5'- TCA CTG CCC CCC **C**TC TCC CGG CTT C -3'  Oligo 5' -TCA CTG CCC CCC **T**TC TCC CGG CTT C -3' | 66ºC |  |
| *MPG* | rs710079 | ch16 | 129223 | F: 5’ - ATC TGC TCC CCA GGT CAT GCA G – 3’  R: 5’ - GGG TGA CCA TCC TGT GGG TTG T – 3’ |  | 60ºC | *Bcc*I |
| Note: PCR-RFLP, polymorphism chain reaction-restriction fragment length polymorphism; CHR, chromosome. | | | | | | | |

| **Table S2.** Information of *NPRL3*, *MPG* gene polymorphisms from WES results | | | |
| --- | --- | --- | --- |
| Gene | Location | Rs number | *P*-value |
| MPG | 5_prime_UTR | rs76079375 | >0.05 |
| MPG | 5_prime_UTR | rs2562162 | 0.00007259 |
| MPG | 5_prime_UTR | . | >0.05 |
| MPG | 5_prime_UTR | . | >0.05 |
| MPG | 5_prime_UTR | rs710079 | >0.05 |
| MPG | 5_prime_UTR | rs3176380;rs2234890 | >0.05 |
| MPG | 5_prime_UTR | rs710080 | >0.05 |
| MPG | synonymous | rs710081 | >0.05 |
| MPG | missense | rs201536549 | >0.05 |
| MPG | intron | . | >0.05 |
| NPRL3 | intron | rs743725 | 0.0002432 |
| NPRL3 | frameshift | rs57321480;rs397815833 | >0.05 |
| NPRL3 | intron | rs559166204 | >0.05 |
| NPRL3 | intron | rs2541618 | 0.0000468 |
| NPRL3 | intron | rs2541616 | >0.05 |
| NPRL3 | intron | rs808892 | 0.0003943 |
| NPRL3 | intron | rs369659219 | >0.05 |
| NPRL3 | intron | rs116993855 | >0.05 |
| NPRL3 | intron | rs148796095 | >0.05 |
| NPRL3 | intron | . | >0.05 |
| NPRL3 | intron | rs138059300 | >0.05 |
| NPRL3 | splice_region & intron | rs61016911 | >0.05 |
| NPRL3 | synonymous | rs75187722 | 0.01976 |
| NPRL3 | splice_acceptor & 5_prime_UTR & intron | . | >0.05 |
| Note: NPRL3, nitrogen permease receptor like-3; MPG, N-methylpurine DNA glycosylase. | | | |

| **Table S3.** Comparison of baseline characteristics between ischemic stroke patients, ischemic stroke subgroups, and controls | | | | | | | | | |
| --- | --- | --- | --- | --- | --- | --- | --- | --- | --- |
| Characteristic | Controls  (n=417) | Stroke patients  (n=519) | *P*^a^ | LAD patients  (n=207) | *P*^a^ | SVD patients  (n=149) | *P*^a^ | CE patients  (n=53) | *P*^a^ |
| BMI (kg/m2, mean ±SD) | 24.37±3.22 | 24.18±3.11 | 0.439 | 24.39±3.07 | 0.931 | 23.98±3.16 | 0.253 | 23.84±3.27 | 0.290 |
| HDL-C (mg/dl, mean ±SD) | 46.39±13.67 | 44.58±15.62 | **0.021^b^** | 43.52±13.26 | **0.039** | 44.03±13.77 | 0.127 | 46.33±13.76 | 0.975 |
| Homocysteine (μmol/L, mean ±SD) | 10.07±4.18 | 11.21±7.34 | **0.005^b^** | 11.40±7.95 | **0.047^b^** | 10.89±5.67 | 0.085^b^ | 9.44±3.75 | 0.294 |
| Folate (nmol/L, mean±SD) | 8.83±7.92 | 7.10±6.15 | **< 0.0001^b^** | 6.40±4.21 | **< 0.0001^b^** | 7.07±5.53 | **< 0.0001^b^** | 9.73±11.53 | 0.309^b^ |
| Vitamin B12 (pg/ml, mean ±SD) | 741.34±662.85 | 752.10±641.72 | 0.803 | 794.03±872.81 | 0.935^b^ | 657.32±314.81 | 0.148^b^ | 813.58±433.98 | **0.013^b^** |
| Total cholesterol (mg/dl, mean ±SD) | 193.12±36.99 | 190.33±40.40 | 0.280 | 193.21±46.28 | 0.682^b^ | 189.32±36.23 | 0.283 | 180.25±34.59 | **0.017** |
| Triglyceride (mg/dl, mean ±SD) | 146.69±89.81 | 152.82±114.81 | 0.761^b^ | 152.10±100.27 | 0.500 | 169.29±125.53 | 0.099^b^ | 134.02±181.59 | **0.007^b^** |
| PLT (10^3^/㎕, mean ±SD) | 242.70±67.84 | 244.71±76.82 | 0.509^b^ | 251.65±84.05 | 0.802^b^ | 239.11±70.40 | 0.584 | 233.34±74.61 | 0.351 |
| PT (sec, mean ±SD) | 11.78±0.80 | 11.82±1.04 | 0.793^b^ | 11.78±0.76 | 1.000 | 11.68±0.79 | 0.199 | 12.07±1.05 | 0.099^b^ |
| aPTT (sec, mean ±SD) | 33.39±18.51 | 30.57±4.78 | **0.043^b^** | 30.49±4.82 | 0.052^b^ | 30.82±4.70 | 0.273^b^ | 30.80±4.27 | 0.760^b^ |
| Fibrinogen (mg/dl, mean ±SD) | 402.27±126.70 | 425.30±127.15 | 0.054 | 431.97±128.17 | 0.224 | 398.40±115.24 | 0.780^b^ | 453.96±133.29 | 0.086 |
| Antithrombin III (%, mean ±SD) | 94.16±43.02 | 93.95±17.39 | 0.078^b^ | 94.82±15.54 | 0.056^b^ | 95.45±20.05 | **0.045^b^** | 86.28±17.23 | 0.130^b^ |
| BUN (mg/dl, mean ±SD) | 15.81±5.00 | 16.14±6.39 | 0.920^b^ | 15.41±4.85 | 0.348 | 15.08±5.14 | 0.135 | 18.68±11.04 | 0.068^b^ |
| Uric Acid (mg/dl, mean ±SD) | 4.64±1.47 | 4.70±1.52 | 0.561 | 4.67±1.42 | 0.811 | 4.62±1.36 | 0.905 | 4.54±1.56 | 0.644 |
| Note: SD, standard deviation; BMI, body mass index; HDL-C, high density lipoprotein cholesterol; PLT, platelet; PT, prothrombin time; aPTT, activated partial thromboplastin time; BUN, blood urea nitrogen; LAD, large artery disease; SVD, small vessel disease; CE, cardioembolism.  ^a^ *P*-values were calculated by two-sided t-test for continuous variables and chi-square test for categorical variables.  ^b^ *P*-values were calculated by Mann-Whitney-test for continuous variables.  ***P*-values< 0,05 are bold** | | | | | | | | | |

| **Table S4.** Stratified analysis of *NPRL3* genotypes and characteristics of ischemic stroke among individual risk factors | | | | | | | | | | | | | | | | |
| --- | --- | --- | --- | --- | --- | --- | --- | --- | --- | --- | --- | --- | --- | --- | --- | --- |
| Characteristics | | *NPRL3* rs2541618 TT | | | | *NPRL3* rs2541618 CT+TT | | | | *NPRL3* rs75187722 GA | | | | *NPRL3* rs75187722 GA+AA | | |
|  |  | AOR(95% CI)^*^ | | *P* | | AOR(95% CI)^*^ | | *P* | | AOR(95% CI)^*^ | | *P* | | AOR(95% CI)^*^ | | *P* |
| Age (936) | |  | |  | |  | |  | |  | |  | |  | |  |
| <63 | | 1.757 (0.853 - 3.620) | | 0.126 | | 1.436 (0.951 - 2.167) | | 0.085 | | 0.605 (0.350 - 1.044) | | 0.071 | | 0.589 (0.343 - 1.014) | | 0.056 |
| ≥63 | | 1.412 (0.710 - 2.810) | | 0.325 | | 1.048 (0.723 - 1.519) | | 0.805 | | 0.762 (0.468 - 1.241) | | 0.275 | | 0.794 (0.493 - 1.281) | | 0.345 |
| Sex (936) | |  | |  | |  | |  | |  | |  | |  | |  |
| Male | | 1.858 (0.913 - 3.782) | | 0.088 | | **1.581 (1.033 - 2.421)** | | **0.035** | | **0.544 (0.310 - 0.957)** | | **0.035** | | **0.544 (0.310 - 0.957)** | | **0.035** |
| Female | | 1.339 (0.673 - 2.667) | | 0.406 | | 1.012 (0.708 - 1.448) | | 0.947 | | 0.815 (0.504 - 1.318) | | 0.404 | | 0.825 (0.517 - 1.316) | | 0.419 |
| Hypertension (936) | |  | |  | |  | |  | |  | |  | |  | |  |
| No | | 1.169 (0.586 - 2.333) | | 0.657 | | 1.060 (0.714 - 1.574) | | 0.773 | | 0.993 (0.598 - 1.650) | | 0.978 | | 0.993 (0.598 - 1.650) | | 0.978 |
| Yes | | **2.109 (1.005 - 4.427)** | | **0.049** | | 1.375 (0.944 - 2.002) | | 0.097 | | **0.529 (0.321 - 0.872)** | | **0.012** | | **0.550 (0.339 - 0.893)** | | **0.016** |
| Diabetes mellitus (936) | |  | |  | |  | |  | |  | |  | |  | |  |
| No | | 1.403 (0.824 - 2.392) | | 0.213 | | 1.154 (0.853 - 1.561) | | 0.353 | | 0.685 (0.454 - 1.033) | | 0.071 | | 0.681 (0.454 - 1.020) | | 0.062 |
| Yes | | 2.782 (0.702 - 11.017) | | 0.145 | | 1.651 (0.863 - 3.160) | | 0.130 | | 0.766 (0.349 - 1.683) | | 0.507 | | 0.838 (0.385 - 1.822) | | 0.656 |
| Hyperlipidemia (936) | |  | |  | |  | |  | |  | |  | |  | |  |
| No | | 1.378 (0.805 - 2.361) | | 0.242 | | 1.180 (0.862 - 1.616) | | 0.301 | | **0.599 (0.394 - 0.909)** | | **0.016** | | **0.594 (0.394 - 0.897)** | | **0.013** |
| Yes | | 3.132 (0.786 - 12.479) | | 0.106 | | 1.367 (0.791 - 2.362) | | 0.262 | | 1.233 (0.574 - 2.651) | | 0.591 | | 1.318 (0.619 - 2.805) | | 0.474 |
| Smoking (930) | |  | |  | |  | |  | |  | |  | |  | |  |
| No | | 1.771 (0.918 - 3.415) | | 0.088 | | 1.225 (0.873 - 1.719) | | 0.241 | | 0.775 (0.495 - 1.216) | | 0.268 | | 0.767 (0.493 - 1.193) | | 0.240 |
| Yes | | 1.504 (0.707 - 3.200) | | 0.289 | | 1.181 (0.745 - 1.871) | | 0.479 | | **0.535 (0.287 - 0.998)** | | **0.049** | | 0.568 (0.307 - 1.052) | | 0.072 |
| HDL-c (671) | |  | |  | |  | |  | |  | |  | |  | |  |
| ≥40(M)/50(F) | | 1.083 (0.445 - 2.634) | | 0.860 | | 0.945 (0.570 - 1.568) | | 0.827 | | 0.510 (0.238 - 1.094) | | 0.084 | | 0.510 (0.238 - 1.094) | | 0.084 |
| <40(M)/50(F) | | 1.354 (0.556 - 3.295) | | 0.504 | | 1.453 (0.877 - 2.405) | | 0.147 | | 0.589 (0.324 - 1.070) | | 0.082 | | 0.623 (0.345 - 1.127) | | 0.118 |
| Folate ^*^ (927) | |  | |  | |  | |  | |  | |  | |  | |  |
| >3.54 nmol/L | | 1.387 (0.808 - 2.381) | | 0.235 | | 1.176 (0.874 - 1.582) | | 0.285 | | 0.917 (0.618 - 1.359) | | 0.665 | | 0.910 (0.617 - 1.341) | | 0.633 |
| ≤3.54 nmol/L | | 2.949 (0.580 - 15.003) | | 0.193 | | 1.408 (0.613 - 3.237) | | 0.420 | | **0.137 (0.048 - 0.386)** | | **0.0002** | | **0.142 (0.051 - 0.399)** | | **0.0002** |
| Homocysteine ^†^ (931) | |  | |  | |  | |  | |  | |  | |  | |  |
| <13.7 μmol/L | | 1.546 (0.917 - 2.605) | | 0.102 | | 1.257 (0.936 - 1.687) | | 0.128 | | 0.736 (0.493 - 1.098) | | 0.133 | | 0.737 (0.497 - 1.093) | | 0.129 |
| ≥13.7 μmol/L | | 1.454 (0.295 - 7.171) | | 0.646 | | 0.930 (0.432 - 2.001) | | 0.852 | | 0.537 (0.217 - 1.329) | | 0.179 | | 0.578 (0.237 - 1.411) | | 0.229 |
| Platelet ^†^ (927) | |  | |  | |  | |  | |  | |  | |  | |  |
| <305 10^3^/㎕ | | 1.375 (0.812 - 2.330) | | 0.236 | | 1.138 (0.847 - 1.531) | | 0.391 | | 0.918 (0.610 - 1.381) | | 0.680 | | 0.906 (0.606 - 1.355) | | 0.630 |
| ≥305 10^3^/㎕ | | 3.847 (0.859 - 17.218) | | 0.078 | | 1.814 (0.880 - 3.742) | | 0.107 | | **0.291 (0.121 - 0.701)** | | **0.006** | | **0.322 (0.137 - 0.757)** | | **0.009** |
| PT ^*^ (801) | |  | |  | |  | |  | |  | |  | |  | |  |
| >11.00 sec | | 1.197 (0.690 - 2.075) | | 0.523 | | 1.157 (0.834 - 1.605) | | 0.382 | | **0.577 (0.381 - 0.874)** | | **0.009** | | **0.560 (0.371 - 0.844)** | | **0.006** |
| ≤11.00 sec | | 3.859 (0.627 - 23.766) | | 0.145 | | 1.853 (0.851 - 4.037) | | 0.120 | | 1.093 (0.365 - 3.280) | | 0.874 | | 1.241 (0.426 - 3.614) | | 0.692 |
| aPTT ^*^ (801) | |  | |  | |  | |  | |  | |  | |  | |  |
| >26.30 sec | | 1.678 (0.928 - 3.035) | | 0.087 | | 1.233 (0.890 - 1.709) | | 0.207 | | **0.585 (0.385 - 0.889)** | | **0.012** | | **0.588 (0.389 - 0.890)** | | **0.012** |
| ≤26.30 sec | | 0.568 (0.164 - 1.973) | | 0.373 | | 1.559 (0.698 - 3.481) | | 0.279 | | 0.744 (0.238 - 2.320) | | 0.610 | | 0.666 (0.223 - 1.988) | | 0.466 |
| Fibrinogen ^†^ (626) | |  | |  | |  | |  | |  | |  | |  | |  |
| <537 mg/dl | | 1.685 (0.784 - 3.621) | | 0.181 | | 1.334 (0.886 - 2.007) | | 0.167 | | 0.692 (0.411 - 1.165) | | 0.166 | | 0.720 (0.429 - 1.208) | | 0.213 |
| ≥537 mg/dl | | 0.948 (0.196 - 4.590) | | 0.947 | | 1.280 (0.413 - 3.966) | | 0.669 | | **0.176 (0.040 - 0.767)** | | **0.021** | | **0.176 (0.040 - 0.767)** | | **0.021** |
| Note: AOR, adjusted odds ratio; 95% CI, 95% confidence interval; HDL-c, high density lipoprotein cholesterol; PT, prothrombin time; aPTT, activated partial thromboplastin time; NPRL3, nitrogen permease receptor like-3. ^*^ The adjusted odds ratio on the basis of risk factors, such as age, gender, hypertension, diabetes mellitus, hyperlipidemia, smoking. *P*-values <0.05 are bold. The number of each subgroup is located next to each subgroup. ^†^ Folate 3.54nmol/L, PT 11.00 sec, and aPTT 26.30 sec were lower 15% cut-off each level in ischemic stroke patients and controls. ^‡^ Homocysteine 13.7 μmol/L, Platelet 305 103/㎕, and fibrinogen 537 mg/dl were upper 15% cut-off each level in ischemic stroke patients and controls. | | | | | | | | | | | | | | | | |
| **Table S5.** Stratified analysis of *MPG* genotypes and characteristics of ischemic stroke among individual risk factors | | | | | | | | | | | | | | | | |
| Characteristics | *MPG* rs2562162 CT | | | | *MPG* rs2562162 CT+TT | | | | *MPG* rs710079  CT | | | | *MPG* rs710079 CT+TT | | | |
|  | AOR(95% CI)^*^ | | *P* | | AOR(95% CI)^*^ | | *P* | | AOR(95% CI)^*^ | | *P* | | AOR(95% CI)^*^ | | *P* | |
| Age (936) |  | |  | |  | |  | |  | |  | |  | |  | |
| <63 | 1.339 (0.876 - 2.045) | | 0.177 | | 1.435 (0.952 - 2.164) | | 0.085 | | 0.938 (0.592 - 1.486) | | 0.784 | | 0.903 (0.573 - 1.424) | | 0.661 | |
| ≥63 | 1.255 (0.835 - 1.885) | | 0.275 | | 1.157 (0.792 - 1.689) | | 0.451 | | 0.950 (0.628 - 1.436) | | 0.808 | | 0.915 (0.612 - 1.367) | | 0.664 | |
| Sex (936) |  | |  | |  | |  | |  | |  | |  | |  | |
| Male | 1.407 (0.899 - 2.203) | | 0.135 | | 1.461 (0.953 - 2.239) | | 0.082 | | 0.931 (0.575 - 1.508) | | 0.771 | | 0.866 (0.540 - 1.388) | | 0.549 | |
| Female | 1.229 (0.839 - 1.800) | | 0.289 | | 1.168 (0.814 - 1.676) | | 0.399 | | 0.924 (0.618 - 1.380) | | 0.698 | | 0.914 (0.618 - 1.352) | | 0.652 | |
| Hypertension (936) |  | |  | |  | |  | |  | |  | |  | |  | |
| No | 1.357 (0.893 - 2.063) | | 0.153 | | 1.415 (0.948 - 2.110) | | 0.089 | | 1.201 (0.780 - 1.847) | | 0.406 | | 1.093 (0.715 - 1.672) | | 0.680 | |
| Yes | 1.191 (0.802 - 1.769) | | 0.387 | | 1.127 (0.774 - 1.640) | | 0.534 | | 0.779 (0.508 - 1.195) | | 0.252 | | 0.798 (0.526 - 1.210) | | 0.288 | |
| Diabetes mellitus (936) |  | |  | |  | |  | |  | |  | |  | |  | |
| No | 1.188 (0.862 - 1.636) | | 0.293 | | 1.184 (0.873 - 1.605) | | 0.277 | | 0.859 (0.614 - 1.202) | | 0.376 | | 0.829 (0.596 - 1.154) | | 0.266 | |
| Yes | 1.875 (0.945 - 3.719) | | 0.072 | | 1.830 (0.954 - 3.512) | | 0.069 | | 1.728 (0.757 - 3.946) | | 0.194 | | 1.570 (0.728 - 3.388) | | 0.250 | |
| Hyperlipidemia (936) |  | |  | |  | |  | |  | |  | |  | |  | |
| No | 1.373 (0.980 - 1.923) | | 0.065 | | 1.340 (0.976 - 1.839) | | 0.071 | | 0.863 (0.608 - 1.226) | | 0.412 | | 0.821 (0.582 - 1.159) | | 0.262 | |
| Yes | 1.108 (0.634 - 1.936) | | 0.719 | | 1.113 (0.646 - 1.917) | | 0.700 | | 1.321 (0.701 - 2.489) | | 0.390 | | 1.309 (0.709 - 2.418) | | 0.389 | |
| Smoking (930) |  | |  | |  | |  | |  | |  | |  | |  | |
| No | 1.283 (0.896 - 1.838) | | 0.174 | | 1.238 (0.880 - 1.741) | | 0.221 | | 1.004 (0.683 - 1.477) | | 0.983 | | 0.952 (0.654 - 1.387) | | 0.799 | |
| Yes | 1.302 (0.800 - 2.119) | | 0.289 | | 1.341 (0.845 - 2.126) | | 0.213 | | 0.795 (0.477 - 1.327) | | 0.380 | | 0.779 (0.469 - 1.292) | | 0.333 | |
| HDL-c (671) |  | |  | |  | |  | |  | |  | |  | |  | |
| ≥40(M)/50(F) | 1.345 (0.790 - 2.289) | | 0.276 | | 1.358 (0.819 - 2.253) | | 0.236 | | 0.997 (0.562 - 1.769) | | 0.992 | | 0.966 (0.547 - 1.706) | | 0.904 | |
| <40(M)/50(F) | 1.311 (0.763 - 2.252) | | 0.328 | | 1.276 (0.765 - 2.127) | | 0.350 | | **0.571 (0.334 - 0.976)** | | **0.040** | | **0.589 (0.347 - 0.999)** | | **0.050** | |
| Folate ^*^ (927) |  | |  | |  | |  | |  | |  | |  | |  | |
| >3.54 nmol/L | 1.277 (0.936 - 1.741) | | 0.123 | | 1.235 (0.917 - 1.663) | | 0.165 | | 1.026 (0.733 - 1.437) | | 0.879 | | 0.969 (0.698 - 1.347) | | 0.852 | |
| ≤3.54 nmol/L | 1.391 (0.538 - 3.598) | | 0.496 | | 1.555 (0.654 - 3.698) | | 0.318 | | 0.635 (0.257 - 1.572) | | 0.326 | | 0.714 (0.293 - 1.738) | | 0.458 | |
| Homocysteine ^†^ (931) |  | |  | |  | |  | |  | |  | |  | |  | |
| <13.7 μmol/L | **1.438 (1.054 - 1.960)** | | **0.022** | | **1.455 (1.083 - 1.956)** | | **0.013** | | 0.909 (0.652 - 1.268) | | 0.573 | | 0.864 (0.624 - 1.195) | | 0.377 | |
| ≥13.7 μmol/L | 0.536 (0.236 - 1.215) | | 0.135 | | 0.496 (0.229 - 1.070) | | 0.075 | | 1.347 (0.590 - 3.076) | | 0.480 | | 1.407 (0.618 - 3.203) | | 0.415 | |
| Platelet ^†^ (927) |  | |  | |  | |  | |  | |  | |  | |  | |
| <305 10^3^/㎕ | 1.159 (0.846 - 1.586) | | 0.358 | | 1.140 (0.846 - 1.535) | | 0.390 | | 1.032 (0.738 - 1.443) | | 0.854 | | 0.996 (0.717 - 1.383) | | 0.980 | |
| ≥305 10^3^/㎕ | **2.137 (1.007 - 4.533)** | | **0.048** | | **2.278 (1.106 - 4.691)** | | **0.026** | | 0.691 (0.317 - 1.505) | | 0.352 | | 0.654 (0.303 - 1.412) | | 0.279 | |
| PT ^*^ (801) |  | |  | |  | |  | |  | |  | |  | |  | |
| >11.00 sec | 1.154 (0.815 - 1.635) | | 0.420 | | 1.121 (0.808 - 1.555) | | 0.496 | | 0.857 (0.598 - 1.229) | | 0.401 | | 0.813 (0.571 - 1.158) | | 0.251 | |
| ≤11.00 sec | 1.976 (0.864 - 4.518) | | 0.106 | | 1.798 (0.812 - 3.980) | | 0.148 | | 1.349 (0.550 - 3.309) | | 0.513 | | 1.349 (0.559 - 3.252) | | 0.506 | |
| aPTT ^*^ (801) |  | |  | |  | |  | |  | |  | |  | |  | |
| >26.30 sec | 1.125 (0.798 - 1.587) | | 0.502 | | 1.121 (0.809 - 1.555) | | 0.493 | | 0.908 (0.630 - 1.307) | | 0.602 | | 0.857 (0.599 - 1.224) | | 0.396 | |
| ≤26.30 sec | 2.228 (0.916 - 5.417) | | 0.077 | | 1.662 (0.731 - 3.775) | | 0.225 | | 0.998 (0.424 - 2.348) | | 0.996 | | 0.969 (0.419 - 2.239) | | 0.941 | |
| Fibrinogen ^†^ (626) |  | |  | |  | |  | |  | |  | |  | |  | |
| < 537 mg/dl | 1.552 (0.999 - 2.413) | | 0.051 | | **1.598 (1.050 - 2.433)** | | **0.029** | | 0.839 (0.535 - 1.314) | | 0.443 | | 0.834 (0.535 - 1.298) | | 0.421 | |
| ≥ 537 mg/dl | 1.314 (0.408 - 4.232) | | 0.647 | | 1.259 (0.419 - 3.778) | | 0.682 | | 0.601 (0.190 - 1.901) | | 0.386 | | 0.601 (0.190 - 1.901) | | 0.386 | |
| Note: AOR, adjusted odds ratio; 95% CI, 95% confidence interval; HDL-c, high density lipoprotein cholesterol; PT, prothrombin time; aPTT, activated partial thromboplastin time; MPG, N-methylpurine DNA glycosylase. ^*^ The adjusted odds ratio on the basis of risk factors, such as age, gender, hypertension, diabetes mellitus, hyperlipidemia, smoking. *P*-values <0.05 are bold. The number of each subgroup is located next to each subgroup. ^†^ Folate 3.54nmol/L, PT 11.00 sec, and aPTT 26.30 sec were lower 15% cut-off each level in ischemic stroke patients and controls.  ^‡^ Homocysteine 13.7 μmol/L, Platelet 305 103/㎕, and fibrinogen 537 mg/dl were upper 15% cut-off each level in ischemic stroke patients and controls. | | | | | | | | | | | | | | | | |

| **Table S6.** Ischemic stroke incidence by interaction analysis between *NPRL3*, *MPG* genotypes and environmental factors | | | | | | | | |
| --- | --- | --- | --- | --- | --- | --- | --- | --- |
| Characteristics | *NPRL3* rs2541618 CC | *NPRL3* rs2541618 CT+TT | *NPRL3* rs75187722 GG | *NPRL3* rs75187722 GA+AA | *MPG* rs2562162 CC | *MPG* rs2562162 CT+TT | *MPG* rs710079 CC | *MPG* rs710079 CT+TT |
| HDL-c (671) |  |  |  |  |  |  |  |  |
| ≥40(M)/50(F) | 1.000 (reference) | 1.089 (0.758 - 1.565) | 1.000 (reference) | **0.508 (0.297 - 0.870)** | 1.000 (reference) | **1.449 (1.007 - 2.085)** | 1.000 (reference) | 1.078 (0.718 - 1.619) |
| <40(M)/50(F) | **4.529 (2.900 - 7.074)** | **6.364 (4.103 - 9.869)** | **5.541 (3.880 - 7.912)** | **3.699 (2.099 - 6.520)** | **5.718 (3.754 - 8.709)** | **7.330 (4.564 - 11.773)** | **6.449 (4.384 - 9.487)** | **3.929 (2.412 - 6.400)** |
| Folate ^*^ (927) |  |  |  |  |  |  |  |  |
| >3.54 nmol/L | 1.000 (reference) | 1.187 (0.883 - 1.596) | 1.000 (reference) | 0.911 (0.619 - 1.340) | 1.000 (reference) | 1.254 (0.932 - 1.686) | 1.000 (reference) | 0.953 (0.687 - 1.322) |
| ≤3.54 nmol/L | **3.235 (1.739 - 6.019)** | **4.866 (2.527 - 9.370)** | **5.427 (3.151 - 9.347)** | 0.696 (0.282 - 1.717) | **3.347 (1.892 - 5.919)** | **5.601 (2.651 - 11.836)** | **3.925 (2.270 - 6.785)** | **2.622 (1.247 - 5.517)** |
| Homocysteine ^†^  (931) |  |  |  |  |  |  |  |  |
| <13.7 μmol/L | 1.000 (reference) | 1.261 (0.940 - 1.691) | 1.000 (reference) | 0.738 (0.499 - 1.092) | 1.000 (reference) | **1.463 (1.088 - 1.965)** | 1.000 (reference) | 0.860 (0.621 - 1.190) |
| ≥13.7 μmol/L | 1.727 (0.976 - 3.056) | **1.839 (1.057 - 3.201)** | **1.639 (1.041 - 2.581)** | 0.857 (0.391 - 1.875) | **2.365 (1.374 - 4.071)** | 1.301 (0.731 - 2.318) | 1.281 (0.797 - 2.059) | 1.747 (0.882 - 3.462) |
| Platelet ^†^ (927) |  |  |  |  |  |  |  |  |
| <305 10^3^/㎕ | 1.000 (reference) | 1.140 (0.849 - 1.530) | 1.000 (reference) | 0.870 (0.585 - 1.296) | 1.000 (reference) | 1.158 (0.861 - 1.557) | 1.000 (reference) | 0.991 (0.716 - 1.373) |
| ≥305 10^3^/㎕ | 0.670 (0.379 - 1.187) | 1.205 (0.711 - 2.042) | 1.170 (0.749 - 1.829) | **0.356 (0.163 - 0.776)** | 0.658 (0.385 - 1.123) | 1.348 (0.770 - 2.357) | 1.033 (0.650 - 1.643) | 0.662 (0.342 - 1.281) |
| PT ^*^ (801) |  |  |  |  |  |  |  |  |
| >11.00 sec | 1.000 (reference) | 1.113 (0.831 - 1.491) | 1.000 (reference) | **0.666 (0.454 - 0.976)** | 1.000 (reference) | 1.182 (0.882 - 1.585) | 1.000 (reference) | 0.900 (0.653 - 1.240) |
| ≤11.00 sec | 1.067 (0.606 - 1.881) | **2.192 (1.241 - 3.872)** | 1.319 (0.855 - 2.035) | 1.579 (0.608 - 4.099) | 1.201 (0.690 - 2.088) | **2.071 (1.164 - 3.682)** | 1.364 (0.858 - 2.170) | 1.564 (0.744 - 3.290) |
| aPTT ^*^ (801) |  |  |  |  |  |  |  |  |
| >26.30 sec | 1.000 (reference) | 1.176 (0.879 - 1.573) | 1.000 (reference) | **0.679 (0.463 - 0.996)** | 1.000 (reference) | 1.215 (0.907 - 1.627) | 1.000 (reference) | 0.910 (0.660 - 1.257) |
| ≤26.30 sec | 1.190 (0.668 - 2.121) | 1.731 (0.971 - 3.086) | 1.273 (0.812 - 1.996) | 1.255 (0.489 - 3.222) | 1.210 (0.696 - 2.105) | 1.832 (0.996 - 3.372) | 1.312 (0.801 - 2.149) | 1.292 (0.648 - 2.579) |
| Fibrinogen ^†^ (626) |  |  |  |  |  |  |  |  |
| < 537 mg/dl | 1.000 (reference) | 1.204 (0.904 - 1.603) | 1.000 (reference) | 0.804 (0.553 - 1.168) | 1.000 (reference) | 1.272 (0.954 - 1.696) | 1.000 (reference) | 0.982 (0.717 - 1.346) |
| ≥ 537 mg/dl | **2.690 (1.276 - 5.670)** | **3.464 (1.698 - 7.065)** | **3.535 (1.972 - 6.335)** | 0.702 (0.223 - 2.209) | **2.926 (1.483 - 5.770)** | **3.460 (1.580 - 7.578)** | **3.481 (1.838 - 6.595)** | 1.716 (0.727 - 4.049) |
| Note: NPRL3, nitrogen permease receptor like-3; MPG, N-methylpurine DNA glycosylase; PT, prothrombin time; aPTT, activated partial thromboplastin time. *P*-values <0.05 are bold. The number of each subgroup is located next to each subgroup.  ^*^ Folate 3.54nmol/L, PT 11.00 sec, and aPTT 26.30 sec were lower 15% cut-off each level in ischmic stroke patients and controls. ^†^ Homocystein 13.7 umol/L, Platelet 305 10^3^/㎕, and Fibrinogen 537 mg/dl were upper 15% cut-off each level in ischmic stroke patients and controls. | | | | | | | | |

| **Table S7.** Clinical variables of ischemic stroke patients and subtypes stratified according to *NPRL3* and *MPG* polymorphisms | | | | | | | | | | | | |
| --- | --- | --- | --- | --- | --- | --- | --- | --- | --- | --- | --- | --- |
| Genotypes | PLT  (10^3^/µL) | | Uric acid  (mg/dL) | | LAD patients Platelets  (10^3^/µL) | | LAD patients Uric acid  (mg/dL) | | LAD patients HDL-c  (mg/dL) | | SVD patients Fibrinogen   (mg/dL) | |
|  | Mean±SD | *P^*^* | Mean±SD | *P^*^* | Mean±SD | *P*^*^ | Mean±SD | *P*^*^ | Mean±SD | *P*^*^ | Mean±SD | *P^*^* |
| *NPRL3* rs2541618C>T |  |  |  |  |  |  |  |  |  |  |  |  |
| CC | 238.37±81.92 | 0.256 | 4.58±1.50 | 0.455 | 245.99±100.50 | 0.682 | 4.46±1.39 | 0.157 | 45.68±16.13 | 0.110 | 393.32±112.43 | 0.103 |
| CT | 249.98±71.92 |  | 4.76±1.57 |  | 255.96±66.07 |  | 4.83±1.50 |  | 41.52±10.56 |  | 387.55±113.65 |  |
| TT | 247.66±75.39 |  | 4.90±1.35 |  | 257.40±75.36 |  | 4.87±1.18 |  | 42.55±8.42 |  | 450.26±120.70 |  |
| Dominant (CC vs CT+TT) | 249.54±72.47 | 0.101 | 4.79±1.53 | 0.114 | 256.27±67.87 | **0.030**^†^ | 4.84±1.43 | 0.055 | 41.76±10.08 | **0.038** | 401.57±117.51 | 0.684 |
| Recessive (CC+CT vs TT) | 247.66±75.39 | 0.761 | 4.90±1.35 | 0.282 | 257.40±75.36 | 0.646^†^ | 4.87±1.18 | 0.444 | 42.55±8.42 | 0.696 | 450.26±120.70 | **0.034** |
| *NPRL3* rs75187722G>A |  |  |  |  |  |  |  |  |  |  |  |  |
| GG | 245.19±78.12 | 0.916 | 4.70±1.49 | **0.028** | 253.51±83.97 | 0.423 | 4.76±1.46 | **0.023**^†^ | 43.31±10.28 | 0.364^†^ | 397.16±118.19 | 0.922 |
| GA | 241.61±70.88 |  | 4.58±1.58 |  | 239.79±85.13 |  | 4.08±0.94 |  | 44.98±26.14 |  | 407.14±93.19 |  |
| AA | 251.75±50.07 |  | 6.65±2.67 |  | - |  | - |  | - |  | 429.00±0.00 |  |
| Dominant (GG vs GA+AA) | 242.11±69.79 | 0.741 | 4.68±1.68 | 0.927 | 239.79±85.13 | 0.152^†^ | 4.08±0.94 | **0.023**^†^ | 44.98±26.14 | 0.364^†^ | 408.60±89.98 | 0.718 |
| Recessive (GG+GA vs AA) | 251.75±50.07 | 0.854 | 6.65±2.67 | 0.098^†^ | - |  | - |  | - |  | 429.00±0.00 | 0.791 |
| *MPG* rs2562162C>T |  |  |  |  |  |  |  |  |  |  |  |  |
| CC | 237.17±76.06 | 0.071 | 4.74±1.50 | 0.710 | 242.25±91.32 | 0.247 | 4.60±1.28 | 0.586 | 43.49±15.36 | 0.858 | 391.96±110.84 | 0.702 |
| CT | 253.07±75.91 |  | 4.63±1.53 |  | 260.11±72.14 |  | 4.78±1.61 |  | 43.24±11.10 |  | 406.78±120.93 |  |
| TT | 249.89±84.18 |  | 4.76±1.54 |  | 269.00±94.22 |  | 4.48±1.24 |  | 45.37±8.72 |  | 383.83±110.54 |  |
| Dominant (CC vs CT+TT) | 252.61±76.97 | **0.022** | 4.65±1.53 | 0.499 | 261.33±75.07 | 0.103 | 4.74±1.56 | 0.875^†^ | 43.55±10.78 | 0.977 | 403.50±119.08 | 0.561 |
| Recessive (CC+CT vs TT) | 249.89±84.18 | 0.675 | 4.76±1.54 | 0.789 | 269.00±94.22 | 0.425 | 4.48±1.24 | 0.607 | 45.37±8.72 | 0.589 | 383.83±110.54 | 0.664 |
| *MPG* rs710079C>T |  |  |  |  |  |  |  |  |  |  |  |  |
| CC | 245.56±78.74 | 0.629 | 4.69±1.48 | 0.937 | 254.09±86.26 | 0.316 | 4.74±1.50 | 0.342 | 43.06±10.48 | 0.406 | 395.93±118.45 | 0.874 |
| CT | 243.76±72.65 |  | 4.71±1.62 |  | 247.13±77.30 |  | 4.49±1.17 |  | 45.11±19.31 |  | 406.91±105.49 |  |
| TT | 217.71±46.82 |  | 4.87±1.54 |  | 132.00±0.00 |  | 3.30±0.00 |  | 30.80±0.00 |  | 429.00±0.00 |  |
| Dominant (CC vs CT+TT) | 242.47±71.71 | 0.684 | 4.72±1.62 | 0.809 | 245.07±78.12 | 0.595^†^ | 4.47±1.17 | 0.227 | 44.83±19.22 | 0.413 | 407.67±103.67 | 0.628 |
| Recessive (CC+CT vs TT) | 217.71±46.82 | 0.350 | 4.87±1.54 | 0.758 | 132.00±0.00 | 0.090^†^ | 3.30±0.00 | 0.336 | 30.80±0.00 | 0.337 | 429.00±0.00 | 0.791 |
| Note: ANOVA, analysis of variance; HDL-c, high density lipoprotein cholesterol; LAD, large artery disease; SVD, small vessel disease; CE, cardioembolism; SD, standard deviation; NPRL3, nitrogen permease receptor like-3; MPG, N-methylpurine DNA glycosylase.  ^*^Calculated using ANOVA. ^†^Calculated using the Kruskal-Wallis test. *P*-values< 0,05 are bold | | | | | | | | | | | | |

| **Table S8.** Results of stepwise Cox regression analysis for ischemic stroke survival | | | | |
| --- | --- | --- | --- | --- |
| Covariate | β | SEM | HR (95% CI) | *P^*^* |
| *MPG* rs2562162 CC vs CT in SVD group |  |  |  |  |
| Age | 0.078 | 0.026 | 1.081 (1.028 - 1.137) | **0.003** |
| *MPG* rs2562162 CC vs CT+TT in SVD group |  |  |  |  |
| Age | 0.078 | 0.024 | 1.082 (1.032 - 1.133) | **0.001** |
| *MPG* rs2562162 CC vs CT+TT in SVD group/with hypertension |  |  |  |  |
| Genetic variant (CC vs CT+TT) | 1.686 | 0.810 | 0.185 (0.038 - 0.899) | **0.037** |
| Age | 0.100 | 0.035 | 1.105 (1.032 - 1.183) | **0.001** |
| *MPG* rs710079 CC vs CT with diabetes mellitus |  |  |  |  |
| Genetic variant (CC vs CT) | 0.788 | 0.381 | 2.200 (1.046 - 4.625) | **0.039** |
| Age | 0.052 | 0.019 | 1.054 (1.016 - 1.093) | **0.005** |
| *NPRL3* rs2541618 CC vs CT+TT with hyperlipidemia |  |  |  |  |
| Genetic variant (CC vs CT+TT) | 1.134 | 0.556 | 3.107 (1.051 - 9.183) | **0.041** |
| Age | 0.076 | 0.019 | 1.079 (1.040 - 1.120) | **<0.001** |
| *MPG* rs710079 CC vs CT in male |  |  |  |  |
| Genetic variant (CC vs CT) | 0.825 | 0.370 | 2.282 (1.109 - 4.696) | **0.026** |
| Age | 0.075 | 0.015 | 1.078 (1.047 - 1.109) | **<0.0001** |
| Note: SEM, standard error of the mean; HR, hazard ratio; CI, confidence interval; NPRL3, nitrogen permease receptor like-3; MPG, N-methylpurine DNA glycosylase; SVD, small vessel disease. ^*^*P*-value calculated by Cox proportional-hazards regression based on stepwise method. *P*-values< 0,05 are bold | | | | |

| **Table S9.** Statistical power to detect various genetic associations in the present case-control study | | | |
| --- | --- | --- | --- |
| Characteristics | Table | AOR (95% CI) | Statistical power (%) |
| *NPRL3* rs75187722 Dominant model | Table 2 (LAD) | 0.599 (0.370 - 0.968) | 51.44 |
| *NPRL3* rs2541618TT | Table 2 (SVD) | 2.406 (1.225 - 4.725) | 54.13 |
| *NPRL3* rs2541618 Dominant model | Table 2 (SVD) | 1.524 (1.019 - 2.279) | 61.58 |
| *NPRL3* rs75187722GA | Table 2 (SVD) | 0.474 (0.261 - 0.860) | 53.77 |
| *NPRL3* rs75187722 Dominant model | Table 2 (SVD) | 0.495 (0.277 - 0.886) | 50.62 |
| *MPG* rs2562162CT | Table 2 (SVD) | 1.569 (1.038 - 2.372) | 78.95 |
| *MPG* rs2562162 Dominant model | Table 2 (SVD) | 1.589 (1.069 - 2.363) | 80.70 |
| Note: AOR, adjust odds ratio; 95% CI, 95% confidence interval. | | | |

| **Table S10.** The results of meta-analysis restricted to Europeans in the MEGASTROKE GWAS loci of *NPRL3* and *MPG* genes polymorphisms | | | | | | | | | | | | | | | | | | | |
| --- | --- | --- | --- | --- | --- | --- | --- | --- | --- | --- | --- | --- | --- | --- | --- | --- | --- | --- | --- |
|  |  |  | Ischemic stroke patients & controls | | | | | LAD patients & controls | | | | SVD patients & controls | | | | CE patients & controls | | | |
| SNP | Chr | Gene | Risk allele | MAF | Effect | StdErr | *P*-value | MAF | Effect | StdErr | *P*-value | MAF | Effect | StdErr | *P*-value | MAF | Effect | StdErr | *P*-value |
| rs2541618 | 16 | NPRL3 | T | 0.142 | 0.019 | 0.015 | 0.203 | 0.146 | 0.007 | 0.038 | 0.855 | 0.141 | 0.078 | 0.035 | **0.024** | 0.144 | 0.025 | 0.029 | 0.400 |
| rs75187722 | 16 | NPRL3 | A | 0.016 | -0.069 | 0.057 | 0.222 | 0.015 | -0.097 | 0.158 | 0.537 | 0.016 | 0.133 | 0.142 | 0.350 | 0.017 | -0.123 | 0.109 | 0.257 |
| rs2562162 | 16 | MPG | T | 0.134 | 0.033 | 0.019 | 0.080 | 0.137 | 0.078 | 0.047 | 0.093 | 0.133 | 0.084 | 0.042 | **0.045** | 0.136 | 0.041 | 0.037 | 0.271 |
| rs710079 | 16 | MPG | T | 0.017 | 0.035 | 0.052 | 0.509 | 0.018 | 0.016 | 0.141 | 0.913 | 0.017 | 0.101 | 0.121 | 0.407 | 0.018 | 0.031 | 0.103 | 0.764 |
| Note: GWAS, genome-wide association study; SNP, single nucleotide polymorphism; Chr, chromosome; MAF, minor allele frequency; StdErr, standard error; NPRL3, nitrogen permease receptor like-3; MPG, N-methylpurine DNA glycosylase; LAD, large artery disease; SVD, small vessel disease; CE, cardioembolism. *P*-values< 0,05 are bold | | | | | | | | | | | | | | | | | | | |
